# *Acidisoma silvae* sp. nov. and *Acidisoma* *cellulosilytica* sp. nov., Two Acidophilic Bacteria Isolated from Decaying Wood, Hydrolyzing Cellulose and Producing Poly-3-hydroxybutyrate

**DOI:** 10.3390/microorganisms9102053

**Published:** 2021-09-28

**Authors:** Sophie Mieszkin, Eva Pouder, Stéphane Uroz, Christelle Simon-Colin, Karine Alain

**Affiliations:** 1 Laboratoire de Microbiologie des Environnements Extrêmes LM2E, Université de Brest, CNRS, Ifremer, IUEM, Rue Dumont d’Urville, F-29280 Plouzané, France; pouder.eva@gmail.com (E.P.); Chritelle.Simon.Colin@ifremer.fr (C.S.-C.); Karine.Alain@univ-brest.fr (K.A.); 2 Centre INRAE-Grand Est-Nancy, Université de Lorraine, INRAE, UMR IAM, 54280 Champenoux, F-54000 Nancy, France; stephane.uroz@inrae.fr

**Keywords:** *Acidisoma cellulosilytica* sp. nov., *Acidisoma silvae* sp. nov., cellulose hydrolysis, poly-3-hydroxybutyrate, decaying oak wood, acidophilic

## Abstract

Two novel strains, HW T2.11^T^ and HW T5.17^T^, were isolated from decaying wood (forest of Champenoux, France). Study of the 16S rRNA sequence similarity indicated that the novel strains belong to the genus *Acidisoma*. The sequence similarity of the 16S rRNA gene of HW T2.11^T^ with the corresponding sequences of *A. tundrae* and *A. sibiricum* was 97.30% and 97.25%, while for HW T5.17^T^ it was 96.85% and 97.14%, respectively. The DNA G+C contents of the strains were 62.32–62.50%. Cells were Gram-negative coccobacilli that had intracellular storage granules (poly-3-hydroxybutyrate (P3HB)) that confer resistance to environmental stress conditions. They were mesophilic and acidophilic organisms growing at 8–25 °C, at a pH of 2.0–6.5, and were capable of using a wide range of organic compounds and complex biopolymers such as starch, fucoidan, laminarin, pectin and cellulose, the latter two being involved in wood composition. The major cellular fatty acid was cyclo C_19:0_*ω8c* and the major quinone was Q-10. Overall, genome relatedness indices between genomes of strains HW T2.11^T^ and HW T5.17^T^ (Orthologous Average Nucleotide Identity (OrthoANI) value = 83.73% and digital DNA-DNA hybridization score = 27.5%) confirmed that they belonged to two different species. Genetic predictions indicate that the cyclopropane fatty acid (CFA) pathway is present, conferring acid-resistance properties to the cells. The two novel strains might possess a class IV polyhydroxyalcanoate (PHA) synthase operon involved in the P3HB production pathway. Overall, the polyphasic taxonomic analysis shows that these two novel strains are adapted to harsh environments such as decaying wood where the organic matter is difficult to access, and can contribute to the degradation of dead wood. These strains represent novel species of the genus *Acidisoma*, for which the names *Acidisoma silvae* sp. nov. and *Acidisoma* *cellulosilytica* sp. nov. are proposed. The type strains of *Acidisoma silvae* and *Acidisoma* *cellulosilytica* are, respectively, HW T2.11^T^ (DSM 111006^T^; UBOCC-M-3364^T^) and HW T5.17^T^ (DSM 111007^T^; UBOCC-M-3365^T^).

## 1. Introduction

Deadwood decomposition plays an essential role in forest ecosystems as it maintains the soil fertility and the physico-chemical balance of the biogeochemical cycles in these ecosystems (e.g., carbon (C) cycle, recycling of nitrogen (N), and micronutrients) [1]. This process is mainly performed by a succession of decomposers including insects, fungi, and bacteria that interact to progressively decompose and recycle the nutrients [2,3,4,5]. It also depends on abiotic environmental factors such as humidity, pH, or temperature that are also known to impact deadwood decomposition rates [6]. Wood consists of complex macromolecules such as cellulose, hemicellulose, and lignin, the latter being particularly recalcitrant to decomposition. Its chemical content is variable and depends on the tree species, the tree compartment studied (branch or trunk), and the type of wood (heartwood or sapwood) [7]. Wood decaying fungi, also known as white-, brown- or soft-rot fungi, according to their capacity to degrade the lignocellulose, have long been considered as the main actors of wood decomposition [8,9]. Indeed, their ability to produce extracellular enzymes and the large propagation of hyphae gives them a clear advantage in decomposing organic matter [10,11,12]. The diversity and the role of bacterial communities on the decomposition of the lignocellulose have been less studied. However, López-Mondéjar et al. [13] had shown that forest soil bacterial isolates were capable of contributing actively to the decomposition of cellulose and hemicellulose through the secretion of hydrolytic proteins such as cellulases, hemicellulases, and other glycosyl hydrolases. Concerning decaying wood, an important biomass and a broad diversity of bacteria have been reported across the wood decomposition process, suggesting that the involvement of bacteria in this process does not seem negligible [2]. Dominance of bacteria belonging to *Proteobacteria*, *Actinobacteria*, *Acidobacteria*, and *Bacteroidetes* has been reported considering different wood species [5,14,15,16,17]. If these phyla are representative of the bacterial communities colonizing deadwood, it is important to note that the related bacterial classes or genera differ according to the tree species, the decay stage, and the type of wood [5,17,18,19,20]. While bacteria are poorly effective at decomposing dead wood compared to fungi, a few studies have shown that they are primarily involved in the decomposition of cellulose and pectin as well as in N fixation [21,22,23,24]. However, the functional potential of bacterial assemblages involved in wood decomposition still needs to be better defined, targeting a larger panel of wood biopolymers and their derivatives.

In this context, we investigated (in a previous study) the functional abilities of bacterial strains isolated from decaying oak wood after a nine-months incubation period [17]. This culture dependent approach demonstrated that the bacteria isolated from decaying oak sapwood and heartwood were characterized by their effectiveness at decomposing cellulose and derivatives. The phylogenetic analysis performed on this collection revealed that the most effective and represented genera involved in this process were *Acidisoma*, *Mucilaginibacter*, and *Novosphingobium*. Among these bacteria, two new strains belonging to the genus *Acidisoma* have been more deeply characterized and are described in this study.

The genus *Acidisoma*, with its two sole representatives, *A. tundrae* DSM 19999^T^ and *A. sibiricum* DSM 21000^T^, has been described by Belova et al. [25]. These strains have been isolated from acidic environments (*Sphagnum* peat (pH 4.5) and peat soil (pH 4.2)) and are psychrotolerant, capable of growing at temperature down to 2 °C. Representatives of the *Acetobacteraceae* family (*Alphaproteobacteria*) to which they belong, have been commonly identified from acidic terrestrial and aquatic environments such as acid mine drainages, acidic hot springs, and acidic bogs [26,27,28,29,30] as well as in forest soils and decaying wood [5,31,32]. The two species of *Acidisoma* already described are Gram-negative, non-motile coccobacilli covered with large polysaccharide capsules. They are strict aerobic chemoorganoheterotrophs, and are catalase and oxidase positive. Their major cellular fatty acid is cyclo C_19:0_*ω*8*c*, their main quinone is Q-10, and their phospholipids consist in phosphatidylcholine, diphosphatidylglycerol, phosphatidylethanolamine and phosphatidylglycerol.

In the present study, we describe two new representatives of the *Acidisoma* genus isolated from decaying oak wood, *A. silvae* HW T2.11^T^ (DSM 111006^T^) and *A.* *cellulosilytica* HW T5.17^T^ (DSM 111007^T^), by providing a phenotypic, chemotaxonomic, phylogenetic, and genomic analysis of these new species, and by placing their properties into an environmental context.

## 2. Materials and Methods

The experimental research procedure is presented in Figure S1.1. Briefly, after isolation of the two strains, it consisted of their taxonomic identification by 16S rRNA phylogenetic analysis and genome sequencing. An exploration of the bacterial genomes and physiological predictions were then performed. Morphological, physiological and metabolic features of the cells were determined as well as their susceptibility to various antibiotics. Finally, chemotaxonomic analyses were performed and polyhydroxyalcanoates (PHAs) were characterized.

### 2.1. Isolation and Deposit in Public Culture Collections

Strains HW T2.11^T^ and HW T5.17^T^, were isolated from the heartwood of decaying oak (*Quercus petraea*). Oak discs have been placed during nine months on the soil surface of the forest experimental site of Champenoux (north-eastern of France; Lat: 48.718420° N; Long: 6.346580° E; Alt: 248 m; 0.5 ha of surface) as described by Mieszkin et al. [17]. Briefly, isolation was performed by spreading serially diluted heartwood sawdust suspensions on 1/10 diluted Tryptic Soy Agar (TSA) medium (Difco’s Tryptic Soy Broth (TSB) 3 g.L^−1^ and agar 15 g.L^−1^) containing cycloheximide (100 µg.L^−1^, final concentration) with a pH adjusted to 5. Both strains were then grown on 1/10 TSA or 1/10 TSB media adjusted to pH 5 during three days at 20 °C, under agitation (250 rpm). Their purity was confirmed by repeated microscopic observations, sequencing of their 16S rRNA gene and genome. Stock cultures were stored at −80 °C in 1/10 TSB medium supplemented with 5% (*v*/*v*) dimethyl sulfoxide (DMSO).

Both strains, *A. silvae* str. HW T2.11^T^ (DSM 111006^T^; UBOCC-M-3364^T^) and *A.* *cellulosilytica* str. HW T5.17^T^ (DSM 111007^T^; UBOCC-M-3365^T^) are available in the public culture collections Deutsche Sammlung von Mikroorganismen und Zellkulturen (DSMZ; https://www.dsmz.de/collection (accessed on 1 July 2020)) and UBO Culture Collection (UBOCC; https://www.univ-brest.fr/ubocc (accessed on 1 July 2020)).

### 2.2. Phylogenetic Analysis

Complete double-strand 16S rRNA gene sequences of both strains were generated from one single colony, as described elsewhere [33]. Pairwise 16S rRNA gene sequence similarity was calculated using global alignment algorithm implemented at the EzTaxon-e server (http://eztaxon-e.ezbiocloud.net/ (accessed on 1 July 2020); [34]. Phylogenetic analysis was performed using the software Seaview version 4.7 [35]. The distance matrix was calculated using the Kimura two-parameters model and the clustering was performed with the neighbor-joining algorithm [36]. The robustness of the inferred topology was assessed by bootstrap analyses based on 1000 replications.

### 2.3. Genome Sequencing, Assembly and Annotation

Genomic DNA of strains HW T2.11^T^ and HW T5.17^T^ was extracted with a standard PCI (Phenol: Chloroform: Isoamyl Alcohol (25:24:1)) protocol, as described elsewhere [37]. Whole genome sequencing of both strains was performed by the Fasteris company (Plan-les-Ouates, Swizerland), using the Illumina MiSeq technology (2 × 150 bp paired-reads; MicroV2 chemistry). Quality control, genome assembly and annotation were performed as described by Allioux et al. [38]. Briefly, quality controls of reads were performed by the sequencing facilities while genomes were assembled into contigs, using default parameters, by using the Shovill pipeline (v1.0.9) with SKESA and SPAdes genome assemblers for strain HW T2.11^T^ and strain HW T5.17^T^, respectively. Genome completeness and potential contamination were controlled with CheckM (v1.1.2-https://ecogenomics.github.io/CheckM/ (accessed on 1 July 2020)). Genomes were annotated with the MicroScope Microbial Genome Annotation and Analysis Platform (MaGe; https://mage.genoscope.cns.fr (accessed on 1 July 2020)) using KEGG and BioCyc databases. The search and annotation of genes involved in the production of PHAs has also been performed with the NCBI’s (National Center of Biotechnology Information) integrated Prokaryotic Genome Annotation Pipeline (PGAP) [39]. Functional annotation of predicted coding DNA sequences (CDSs) was checked with NCBI (v2.10.0+) and UniProtKB databases (release 2020_02). The average nucleotide identity score (ANI; OrthoANI value) between the genomes of the two new strains (*NB:* the genomes of the reference strains *A. sibiricum* and *A. tundrae* are not sequenced), was obtained using the ANI calculator tool from the EzBioCloud web server (https://www.ezbiocloud.net/tools/ani (accessed on 1 July 2020)). Digital DNA-DNA hybridization (dDDH) scores between the genomes of strain HW T2.11^T^ and strain HW T5.17^T^, were also determined by the genome-to-genome distance calculator (GGDC 2.1), using formula 2 [40].

### 2.4. Morphological, Physiological and Metabolic Features

Cell morphology and motility were determined by light microscopy (Olympus BX60 and CX40), scanning electron microscopy (SEM; FEI Quanta 200) and transmission electron microscopy (TEM; JEOL JEM 1400). Gram-staining was determined using standard procedures and confirmed with a KOH (3%) test. Catalase and cytochrome oxidase activities were evaluated using H_2_O_2_ and strip of *N,N,N’,N’*-tetramethyl-*p*-phenyldiamine dihydrochloride (Biorad), respectively.

Unless stated otherwise, physiological assays were carried out aerobically in 1/10 TSA medium adjusted to pH 5, under agitation (250 rpm), at 20 °C. Cells were routinely counted by direct cell counting using a modified Thoma chamber (Preciss, France; surface: 0.0025 mm^2^, depth: 0.100 mm). Determination of the temperature range for growth was carried out at −5, 0, 5, 10, 15, 20, 25, 30, 35, 40, and 45 °C for 10 days. The pH range for growth was tested from pH 1.5 to pH 9.0 (at 20 °C), with increments of 0.5 to 1 unit (pH 1.5, 2, 3, 4, 5, 5.5, 6, 6.5, 7, 8, and 9) on 1/10 TSB medium for 10 days. For this experiment, we used the following buffers (each at 20 mM, Sigma-Aldrich, Darmstadt, Germany): for pH 1.5, 2.0 and 3.0, no buffer; for pH 4.0 and 5.0, HOMOPIPES buffer; for pH 5.5–6.5, MES buffer; for pH 7.0, PIPES buffer; for pH 7.5–8.0, HEPES buffer; for pH 8.5, TAPS buffer; and for pH 9.0, CAPSO buffer. Salt tolerance was tested with various concentrations of NaCl (0, 0.5, 2.5, 5, 7.5, and 10% NaCl (*w*/*v*), over a period of 10 days on 1/10 TSA (at 20 °C and pH 5). Optimal growth conditions were defined on the basis of the temperature, pH and salinity optima set in the previous experiments. Growth kinetics were then carried out in triplicates, on TSB 10% medium, containing 0.5% (*w*/*v*) NaCl, adjusted to pH 3.5 for strain HW T2.11^T^ and pH 4.5 for strain HW T5.17^T^, at 20 °C, under agitation (250 rpm), for 4 days, with cell counts every 3 h, in order to determine the growth rate and doubling time of the two strains.

Metabolic features were estimated for the two novel strains (HW T2.11^T^ and HW T5.17^T^) as well as for the two previously described strains (*A. tundrae* and *A. sibiricum*). Oxidative and fermentative utilization of carbohydrates as well as enzymatic activities were evaluated by using the API^®^20NE kit (BioMérieux) according to the manufacturer’s instructions with slight modifications (Mac Farland index = 1; seven days of incubation at 20 °C). Hydrolysis of organic substrates as sole C source was performed on a large panel of substrates. Briefly, each strain was cultivated in 1/10 TSB medium adjusted to pH 5 for 48 h at 20 °C. After three steps of bacterial cells washing using sterile distilled water, the bacterial suspension was adjusted to obtain a Mac Farland index = 1. The hydrolysis of pectin, laminarin and fucoidan and the use of lactate, butyrate, pyruvate, benzoate, lactose, glucose, mannose, sucrose, trehalose, ethanol and asparagine was then determined on the mineral basis of the 1/10 TSB medium (0.5 g NaCl and 0.25 g KH_2_PO_4_) adjusted at pH 5, each substrate supplied at a final concentration of 20 mM, after 14 days of incubation under agitation (250 rpm), at 20 °C. Positive controls were performed for all tests and a medium without C source was used as negative controls. The hydrolysis of cellulose was determined using carboxymethylcellulose (CMC) solid medium composed (per liter) of: 5 g CMC (Sigma), 1 g K_2_HPO_4_, 1 g (NH_4_)_2_SO4, 0.5 g MgSO_4_,7H_2_O, 0.5g NaCl and 20 g agar. The cellulolytic activity was revealed by flooding the plates with a Red Congo (RC) solution (RC diluted in distilled water at 1 mg.mL^−1^) for 40 min. After removal of the RC stain, the plates were washed with 1 M NaCl for 15 min. Strains presenting a positive activity were characterized by the formation of a halo on the plates after the staining and washing treatments. Liquid culture media or agar slants were also used to evidence (i) mannitol fermentation, mobility, and presence of nitrate reductase (Mannitol-Mobility-Nitrate agar medium (MMN) composed (per liter) of 10 g tryptic hydrolysate of casein, 1 g potassium nitrate, 7.5 g mannitol, 40 mg phenol red and 3.5 g agar), (ii) fermentative pathways (mixed acids or butane-2,3-diol pathways; Clark and Lubs liquid medium composed (per liter) of 7 g peptone from meat, 5 g D(+) glucose and 5 g phosphate buffer), (iii) glucose and lactose fermentation, gas and H_2_S production (Kigler-Hajna agar medium composed (per litter) of 10 g casein peptone, 1 g glucose, 10 g lactose, 3 g meat extract, 10 g meat peptone, 3 g yeast extract, 0.025 g phenol red, 5 g sodium chloride, 0.5 g sodium thiosulfate, 0.2 g ferrous sulfate, 0.2 g iron(II) sulfate heptohydrate (Sigma) and 12 g agar), and, (iv) citrate utilization (Simmons citrate agar medium composed (per liter) of 1 g dipotassium phosphate, 5 g sodium chloride, 1 g ammonium dihydrogen phosphate, 0.2 g magnesium sulfate, 2 g sodium citrate, 0.08 g bromothymol blue and 15 g bacteriological agar). For these assays, cultures, washings and suspensions of bacterial cells were prepared as described above.

### 2.5. Susceptibility to Antibiotics

Antibiotic susceptibility of both isolates was determined, in duplicates, using a disc diffusion method. Briefly, the strains were grown in 1/10 TSB medium adjusted to pH 5 and cells were then harvested for dilution in sterilized distilled water to achieve a turbidity of 1.5 McFarland standard. Plates of 1/10 TSA medium pH 5 were then flooded with 1.5 mL of cell suspension to allow a mat growth. Discs impregnated with kanamycin (40 µg), rifampicin (30 µg), novobiocin (30 µg), gentamicin (15 µg), ampicillin (10 µg), penicillin (10 U), oxacillin (5 µg), oxytetracyclin (30 µg), chloramphenicol (30 µg), and streptomycin (10 µg) were then placed on the agar surface. The inhibition zones were read after three days of incubation at 20 °C.

### 2.6. Chemotaxonomic Analyses

In order to analyze respiratory quinones, polar lipids, and fatty acids, cells of strains HW T2.11^T^ and HW T5.17^T^, grown in 1/10 TSB pH 5 at 20 °C under agitation (250 rpm), were harvested by centrifugation (800× *g*; 10 min) at the end of the exponential phase of growth. These analyses were carried out by the Identification Service of the DSMZ (Braunschweig, Germany) as described by Tindall [41,42] and Kuykendall et al. [43].

### 2.7. Polyhydroxyalcanoates (PHAs) Production and Characterization

Production of PHAs by strains HW T2.11^T^ and HW T5.17^T^ was performed in two steps as previously described by Wecker et al. [44] with slight modifications according to the culture conditions of the strains. The first step, to produce biomass, was performed using 800 mL of 1/10 TSB medium adjusted to pH 5, during 48 h, at room temperature under 250 rpm agitation. Cells were then harvested by centrifugation at 7100× *g* during 20 min at 15 °C and then used for the second step to produce PHAs. This step was performed using TSB medium depleted in N with an excess of glucose as sole source of C (10 g/L). The growth conditions were three days at 20 °C and 200 rpm agitation. Cells were then pelleted by centrifugation at 800× *g* for 10 min and lyophilized. Characterization of PHAs was performed as described elsewhere [45]. Briefly, lyophilized cell pellets were ground and PHAs were extracted with chloroform (Chloroform/water; 30%/70%; (*v*/*v*)) for 4 h at 50 °C. Then, a second extraction with water was carried out on the PHAs-containing chloroform phase to remove residual solid particles. After organic phase evaporation, the dry crude extracts were preserved for the following analyses. Due to the low amount of PHAs produced by both isolates, crude extracts were directly analyzed by Fourier transform infrared spectroscopy (FTIR) and nuclear magnetic resonance (NMR).

## 3. Results and Discussion

### 3.1. Phylogenetic Affiliation of Strains HW T2.11^T^ and HW T5.17^T^

**Figure 1:**
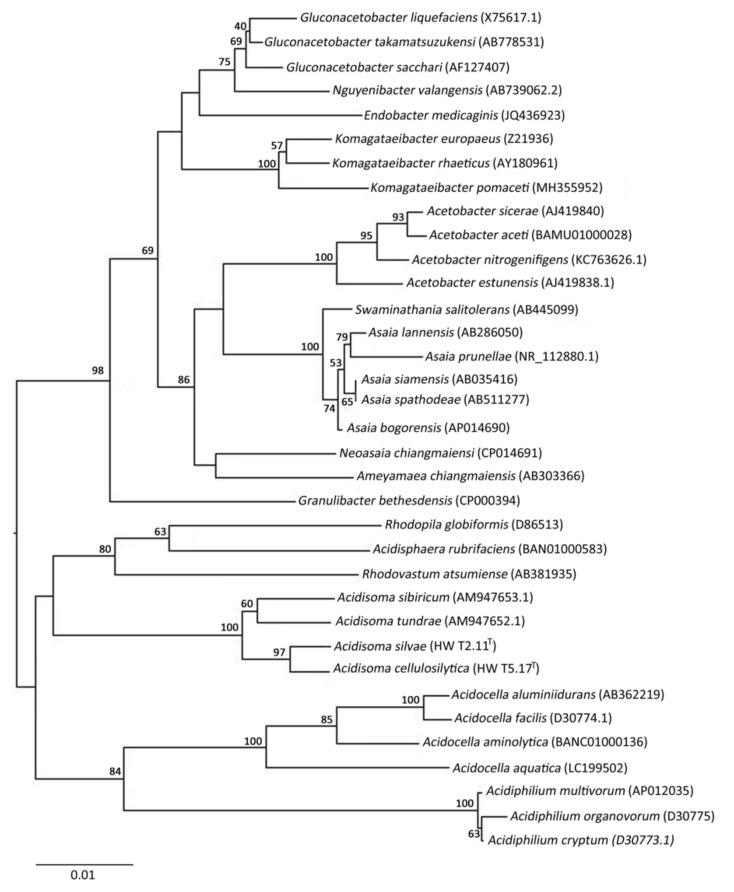
Phylogenetic tree showing the phylogenetic position of strains HW T2.11^T^ (*Acidisoma silvae*) and HW T5.17^T^ (*A.* *cellulosilytica*) and representatives of some other related taxa, based on 16S rRNA gene sequences. The tree was built using the neighbor-joining method and bootstrap values (based on 1000 replicates) are indicated when nodes >50%. For each strain, GenBank accession numbers are given in brackets. The 16S rRNA gene sequence of *Rhodospirillum rubrum* (GenBank D30778) was used as an outgroup (not shown). The scale bar represents 0.01 substitutions per nucleotide position.

Comparative analysis of the 16S rRNA gene sequences of strains HW T2.11^T^ and HW T5.17^T^ showed that they belong to the class *Alphaproteobacteria*, the family *Acetobacteriaceae* and the genus *Acidisoma*. In the neighbor-joining tree based on 16S rRNA gene sequences, strains HW T2.11^T^and HW T5.17^T^ formed a distinct clade with *A. tundrae* and *A. sibiricum* strains (Figure 1). The 16S rRNA gene sequence similarity of HW T2.11^T^ with corresponding sequences of *A. tundrae* and *A. sibiricum* was 97.30% and 97.25%, respectively, while for HW T5.17^T^ it was of 96.85% and 97.14%. 16S rRNA gene sequences of strains HW T2.11^T^ and HW T5.17^T^ were more distantly related to sequences of acidophilic and phototrophic strains belonging to the genera *Acidisphaera* (94.12–94.79%), *Rhodovastum* (92.83–93.38%), *Rhodopila* (92.15–93.71%) and *Acidiphilum* (92.29–92.56%). They were also distantly related to 16S rRNA gene sequences of acidophilic strains belonging to the genus *Acidocella* (92.18–93.78%). The level of 16S rRNA gene sequence similarity with the type strains of *Acidisoma* showed that both novel isolates displayed sufficient molecular differences for a delineation at the species level as these values were below the threshold value (98.70–99%) [46]. The 16S rRNA gene sequence similarity between sequences of strains HW T2.11^T^and HW T5.17^T^ was 98.39%, suggesting that these strains represent also two different species (Figure 1).

### 3.2. Morphological and Biochemical Characteristics of Strains HW T2.11^T^ and HW T5.17^T^

**Figure 2:**
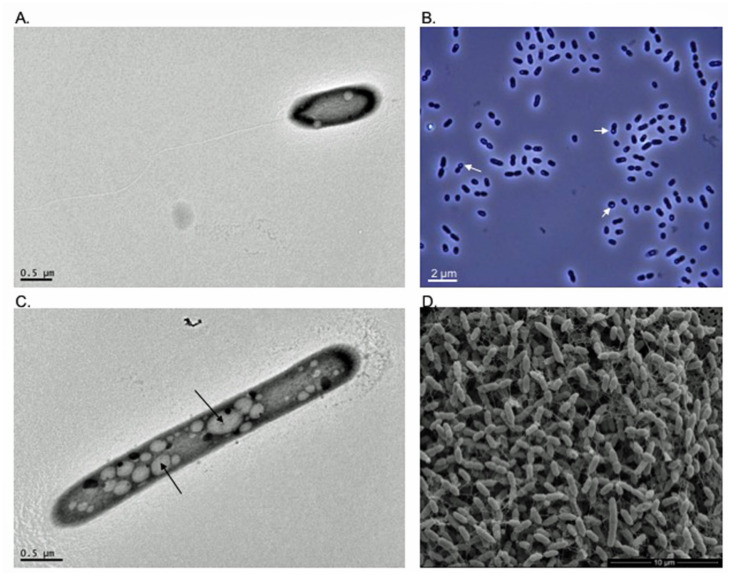
(**A**) Transmission electron micrograph of a cell of strain HW T2.11^T^ (*Acidisoma silvae*) with a polar flagellum; (**B**) phase contrast micrograph of cells of strain HW T2.11^T^ showing clear refractive intracellular granules of poly-*β*-hydroxybutyrate (PHB). White arrows indicate granules of PHB; (**C**) transmission electron micrograph of a cell of strain HW T2.11^T^ with intracellular granules of PHB. Black arrows indicate granules of PHB; (**D**) scanning electron micrograph of cells of strain HW T5.17^T^ (*A.* *cellulosilytica*) showing filamentous structures connecting cells and cells dividing by constriction.

Under optimal growth conditions, colonies of strains HW T2.11^T^ and HW T5.17^T^ are circular with regular edges and smooth surface. Their color is between white and pearly. Cells of strains HWT2-11^T^ and HWT5-17^T^ are Gram-negative coccobacilli dividing by binary fission, that could occur singly, or frequently grouped in pairs or chains. Both strains are catalase positive and oxidase negative. This latter feature is a major difference from *A. tundrae* (WM1^T^) and *A. sibiricum* (TPB606^T^) strains that exhibit cytochrome oxidase activity [25]. Strain HW T2-11^T^ is motile (Figure 2A) while no motility was observed for strain HWT5-17^T^ by light and electronic microscopy and using MMN agar medium. Both strains often formed pellets in liquid medium. During the exponential phase of growth, cell size ranged from 0.485 to 1.041 μm wide (mean 0.765 μm; *n* = 43) and 0.836 to 3.714 μm long (mean 1.809 μm; *n* = 43) for strain HW T2.11^T^, and from 0.60 to 1.014μm wide (mean 0.743 μm; *n* = 52) and 0.952 to 3.014 μm long (mean 1.807 μm; *n* = 52) for HW T5.17^T^ cells. Intracellular production of clear refractive granules was observed at suboptimal growth pHs, or in N-deficient glucose liquid medium, as previously observed with cells of *A. tundrae* (WM1^T^) and *A. sibiricum* (TPB606^T^) [25] (Figure 2B,C). To confirm the potential production of polyhydroxyalcanoates (PHAs), chemical characterization of these granules was performed in the present study. Nanotubes-like filamentous structures connecting cells of strain HW T5.17^T^ were also observed by SEM (Figure 2D). Intercellular nanotubes are known to allow the transport of molecules such as metabolites, proteins or non-conjugative plasmids between cells and could be also involved in cell disintegration [47,48,49].

Strains HW T2.11^T^ and HW T5.17^T^ grow at temperature ranges of 8–25 °C and 14–25 °C, respectively, with an optimum at 20 °C for both strains (Table 1). In comparison, *A. tundrae* (WM1^T^) and *A. sibiricum* (TPB606^T^) grow at lower temperatures (i.e., 2 °C) but have optimal growth around 20 °C [25], indicating that these four strains are all mesophilic. Cells of strains HW T2.11^T^ and HW T5.17^T^, grow, respectively, from pH 2.0 to 6.5 and from pH 3.0 to 5.5 with respective pH optima at pH 3.0–4.0 and pH 4.0–5.0. Both novel isolates are thus as acidophilic to even more acidophilic than *A. tundrae* and *A. sibiricum* which do not grow below pH 3.0 and 3.7, respectively [25]. Their growth is inhibited by NaCl concentrations above 2.5% and their optimal growth is obtained with 0.5% NaCl (Table 1). These results are similar to those obtained by Belova et al. [25], except for *A. tundrae* (WM1^T^) which was not able to grow with more than 1.5% NaCl. Under optimal growth conditions, the generation time of strains HW T2.11^T^ and HW T5.17^T^ is similar (4 h), and much shorter than that described for *A. tundrae* and *A. sibiricum* (6–10 h) (Figure S1.2) [25].

The novel isolates are both chemoorganoheterotrophs, growing by aerobic respiration and using a wide range of organic substrates, and notably carbohydrates, amino acids, and carboxylic acids (only for HW T5.17^T^) (see Table 1 and Table S1.1 for details). As observed for *A. tundrae* and *A. sibiricum*, strains HW T2.11^T^ and HW T5.17 ^T^ catabolize sorbitol, inositol, sucrose, glycerol, yeast extract, and casamino acids. Pyruvate is only used by *A. tundrae* and *A. sibiricum* strains while HW T2.11^T^ is not capable of using glucose as a sole C source. The two novel strains, isolated from decaying wood, are also able to hydrolyze different polymers such as CMC, pectin, fucoidan, laminarin, and Tween 80 (the latter, only for HW T5.17^T^). However, they do not degrade xylan and chitin (Table S1.1; Mieszkin et al. [17]). Under anaerobic conditions using 1/10 TSB medium at pH 5, a weak growth was observed for strains HW T2.11^T^ and HW T5.17^T^_._ Concerning the enzymatic activities, only the strain HW T2.11^T^ has the urease as observed for *A. sibiricum* and is capable of using nitrite or nitrate as terminal electron acceptors revealing the presence of the nitrate reductase. The arginine dihydrolase was active in the two new species but not detected in *A. tundrae* and *A. sibiricum* (Table 1). In addition, lysin and ornithin decarboxylases were evidenced in the two novel strains (data not shown). No fermentative pathway (mixed acids or butane-2,3-diol; pyruvate) was evidenced for the two new strains nor for *A. tundrae* and *A. sibiricum.* None of the strains was capable to produce gas or H_2_S.

Both novel strains, HW T2.11^T^ and HW T5.17^T^, are resistant to chloramphenicol (30 µg), penicillin (10 U), oxacillin (5 µg), and ampicillin (10 µg) while they are sensitive to streptomycin (10 µg), oxytetracycline (30 µg), kanamycin (40 µg), rifampicin (30 µg), and novobiocin (30 µg). Only HW T2.11^T^ was susceptible to gentamicin (15 µg).

The chemotaxonomic markers of the new isolates are broadly similar to those of the other two strains of the genus. Ubiquinone (Q-10) is the main respiratory quinone in strains HW T2.11^T^ and HW T5.17^T^, as described for *A.* *tundrae* and *A. sibiricum* [25]. Low amounts of Q-8 (0.3–0.6%), Q-9 (2.9–4.5%) and Q-11 (0.2–0.11%) were also detected in both novel isolates. The polar lipid patterns of strains HW T2.11^T^ and HW T5.17^T^ were relatively similar and consisted of diphosphatidylglycerol, phosphatidylglycerol, phosphatidylcholin, phosphatidylethanolamine, an unknown aminolipid and an unknown phospholipid. Phosphatidylserin was only identified in strain HW T5.17^T^. All of these polar lipids have been reported in the two other strains of the *Acidisoma* genus. Cellular fatty acid composition of both strains is given in Table 2. The main fatty acids detected in strains HW T2.11^T^ and HW T5.17^T^ were cyclo C_19: 0_*ω8c* (53.86% and 55.85% of the total amount of fatty acids, respectively), C_18: 1_*ω7c,* C_18: 0_ 3-OH and C_16: 0_, as previously described for *A. tundrae* and *A. sibiricum* [25]. However, a notable difference in fatty acid composition was observed in the amount of C_18: 1_ 2-OH, which was high for both strains HW T2.11^T^ (5.9%) and HW T5.17^T^ (8.8%), but low in *A. sibiricum* (0.8%) cells, and absent in *A. tundrae* cells (Table 2; Belova et al. [25]). This difference could be explained by the different culture medium that was used to prepare the biomass of the two novel strains (1/10 TSB against MB medium). The presence of C_18: 1_*ω7c* together with Q-10 is typical of the vast majority of taxa within the *Alphaproteobacteria*.

### 3.3. PHAs Characterization

**Figure 3:**
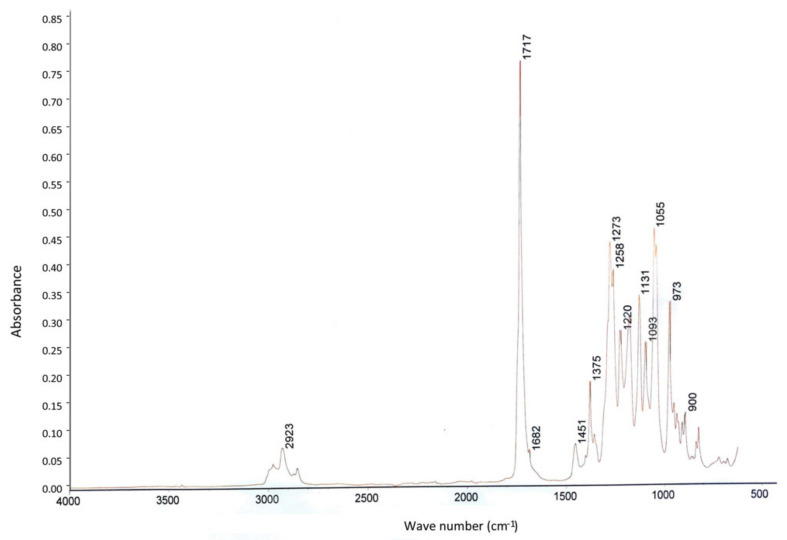
FTIR spectrum of PHAs produced by HW T5.17^T^ strain (*Acidisoma cellulosilytica*) grown on TSB depleted in nitrogen and supplemented with glucose as sole carbon source (a similar spectrum was obtained with strain HW T2.11^T^).

Similar FTIR and ^1^H NMR spectra were obtained for HW T2.11^T^ and HW T5.17^T^ strains, indicating the production of the same biopolymer as an intracellular C and energy storage material when the cells use glucose as sole C substrate. Typical features of short-chain length PHA (*scl*-PHA, 3 to 5 C monomers) and especially poly-3-hydroxybutyrate (P3HB) were observed on spectra. FTIR spectra of PHA crude extracts showed the typical structure of a polyester with an intense absorption band at 1717 cm^−1^, corresponding to ester carbonyl group (C=O) stretching band. The bands near 2923 cm^−1^ are assigned to symmetric and anti-symmetric stretching of the aliphatic C-H, and the one at 1375 cm^−1^ is related to the deformation vibration of the terminal methyl groups (Figure 3).

**Figure 4:**
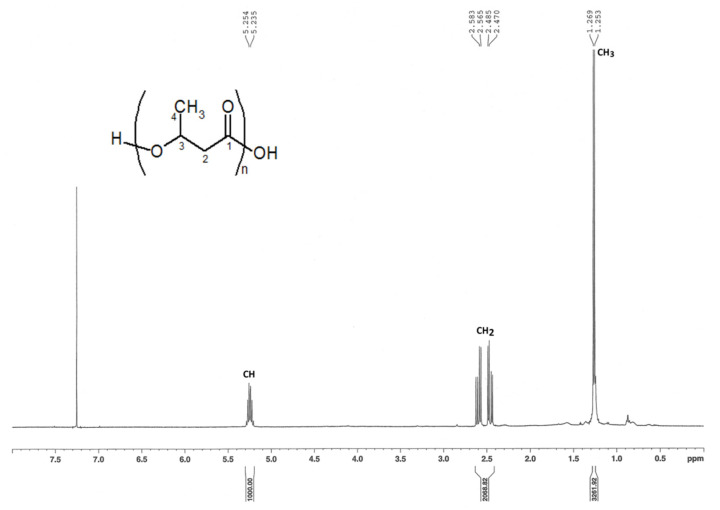
^1^H NMR spectrum of PHAs produced by HW T5.17^T^ strain (*Acidisoma cellulosilytica*) grown on TSB depleted in nitrogen and supplemented with glucose as sole carbon source (a similar spectrum was obtained with strain HW T2.11^T^).

Furthermore, from the ^1^H NMR spectra, the signal at 5.2 ppm was assigned to methine protons (CH) at 5.2 ppm, multiplet resonance between 2.4 and 2.6 ppm corresponded to the methylene protons (CH_2_) of C-2 carbon atom, and the signal at 1.26 ppm was related to the terminal methyl group (CH_3_) in C-4 position as described already by Simon-Colin et al. [45] (Figure 4). Overall, these results confirmed the production of *scl*-PHAs containing 3-hydroxybutyrate units by HW T2.11^T^ and HW T5.17^T^ strains as previously observed for *A. tundrae* and *A. sibiricum* strains [25]. P3HB granules can be of interest as bio-derived and biodegradable plastics but show limits in their mechanical properties due to their rigidity in contrast to medium- or long-chain length PHAs [44]. Such biopolymers have been also extensively studied for their biotechnological applications. PHAs accumulation by bacteria has been proposed to endow microorganisms with stress tolerance under extreme conditions, especially to improve competition abilities of cells exposed to salinity, thermal and oxidative stress, UV radiation, desiccation, and osmotic pressure [50,51,52].

### 3.4. Genome Analysis of Strains HW T2.11^T^ and HW T5.17^T^

#### 3.4.1. General Genome Features

The overall genome size of strain HW T2.11^T^ is 5,444,225 bp. Its draft genome consists in 82 contigs and has a GC content of 62.50%. It was estimated to be 100% complete. The genome size of strain HW T5.17^T^ is 6,010,181 bp (for 99.3781% completion), consisting in 59 contigs and with a GC content of 62.32% (Table 3). Genomic features such as longest contig, *N_50_* and *L_50_* values are given in Table 3. Annotations with MaGe resulted in the prediction of 5501 and 6045 coding DNA sequences (CDSs) for HW T2.11^T^ and HW T5.17^T^, respectively. The genome of str. HW T2.11^T^ contains one *rrn* operon (16S-23S-5S), while the genome of str. HW T5.17^T^ encodes two *rrn* operons, as also confirmed with the Barrnap software. A higher number of tRNA was predicted in the genome of HW T5.17^T^ (52 tRNA) than in the genome of HW T2.11^T^ (45 tRNA). The tRNA found in both cases corresponded to the 20 essential amino acids (Table 3; Figure S1.4a,b).

Most of the CDSs of both genomes could be assigned to at least one cluster of orthologous groups (COGs). Indeed, 5296 CDSs for HW T2.11^T^ (96.3%) and 5864 CDSs for HW T5.17^T^ (97%) were assigned to a COG functional category (Table S1.2). COGs categories related to metabolic processes were dominant (44.30% and 46.45% of the CDSs for HW T2.11^T^ and HW T5.17^T^, respectively). The main categories (>5% of the CDSs) were (i) amino acid transport and metabolism (E, 11.42% and 11.5% of the CDSs for HW T2.11^T^ and HW T5.17^T^, respectively), (ii) carbohydrate transport and metabolism (G, 9.45% and 11.05%), (iii) inorganic ion transport and metabolism (P, 6.02% and 6.04%) and iv) energy production and conversion (C, 5.05% and 5.13%). For both strains, a similar number of CDSs were predicted to be involved in the processes dedicated to the information storage and processing (16.3% of the CDSs). Finally, respectively 16.23% and 14.31% of the assigned CDSs of HW T2.11^T^ and HW T5.17^T^ were allocated to cellular processes and signaling (M; 5.33% and 4.91%) (Table S1.2). Additionally, both genomes contained several integrases and transposases suggesting that both strains have a certain genomic plasticity.

#### 3.4.2. Overall Genome Relatedness Indices (OGRI)

The genomes of both novel isolates shared an OrthoANIu value of 83.73%, which is far below the ANI value of 95–96% generally accepted as a boundary for species delineation [53]. Digital DNA-DNA hybridization score was also well below the DDH threshold level for species demarcation (70%) [54], with a value of 27.5%, confirming that strains HW T2.11^T^and HW T5.17^T^ represent two new genomic species. Overall, genomes relatedness indices confirmed that strains HW T2.11^T^ and HW T5.17^T^ belong to two different species. A global survey of the occurrence of the genus *Acidisoma* in public gene libraries and metagenomes, using the GBIF application, revealed 4423 occurrences, including 4156 geo-referenced data, worldwide, indicating that this genus is very widely distributed (https://www.gbif.org/species/8071706 (accessed on 1 July 2020)) (Figure S1.3). The main habitat where the presence of this genus has been reported is the soil but it has also been identified, in a lesser extent, in geothermal, marine or fluvial ecosystems, suggesting that cells of *Acidisoma* could have a potential for adaptation to varied environments with a wide range of physico-chemical parameters.

#### 3.4.3. Central Metabolism and Energetic Pathways

Based on genome analyses, both strains possess complete pathways for the biosynthesis of the following 20 amino acids: alanine, arginine, cysteine, homocysteine, glutamate, L-glutamine, glycine, histidine, leucine, isoleucine, lysine, ornithine, phenylalanine, proline, serine, homoserine, threonine, tryptophan, tyrosine, and valine. They also contain the metabolic pathways for organoheterotrophic growth on carbohydrates, peptides, amino acids, and some polymers. Notably, they possess the full glycolysis pathway, the non-oxidative branch of the pentose phosphate pathway, the neoglucogenesis path, and the degradation pathways for several amino acids (aspartate, asparagine, alanine, glutamate, glutamine, glycine, histidine, cysteine, serine, proline, taurine, and threonine) and numerous compounds such as ethanol, butanediol, glycerol, choline, and urea (only in *A. silvae* (strain HW T2.11^T^) for the latter). The urease activity for this strain was experimentally validated in the present study. The tricarboxylic acid cycle (TCA) pathway for aerobic respiration was complete in both genomes. The cytochrome C oxidase complex was also identified in both genomes despite that the oxidase activity was not demonstrated experimentally. Both genomes possess all the genes required for pyruvate fermentation pathway to lactate, but this metabolism was not demonstrated under our experimental conditions of anaerobic culture. In addition, the exploration of the genomes predicted that only HW T2.11^T^ is capable of performing pyruvate fermentation to acetate or ethanol. The *Bifidobacterium* shunt pathway leading to acetate, lactate, and energy production was predicted only in the genome of strain HW T5.17^T^ [55]. The pyruvate oxidation pathway, which yields acetate and CO_2,_ through the action of the pyruvate oxidase (PoxB) was identified for HW T2.11^T^ and may contribute to the enhancement of aerobic growth of this strain [56]. Other signaling pathways, conferring competitive advantages in forest ecosystem have also been identified. The cyclopropane fatty acid (CFA) biosynthesis pathway was also present in both genomes. This pathway is responsible for the post-synthetic modification of the lipid bilayer of bacteria entering stationary phase to confer acid resistance properties [57]. This is consistent with the native environment (i.e., decaying oak wood) of the strains HW T2.11^T^ and HW T5.17^T^, where the pH ranged from 3.75 to 4.63 [17]. Although both strains have in their genome the nitrate VIII reduction pathway (dissimilatory) for anaerobic nitrate respiration, this anaerobic respiration has been shown experimentally only for strain HW T2.11^T^. The ammonium transport and phosphate acquisition pathways were also present in both genomes.

#### 3.4.4. Enzymes Involved in Cellulose Hydrolysis

Interestingly, based on genes homology using PGAP and MaGe pipelines, enzymes involved in cellulose degradation have been identified for both strains, confirming the experimental results revealing the capacity of these novel strains to hydrolyze cellulose (Table S1.3). Based on PGAP annotations, one CDS encoding for a cellulase was identified for HW T2.11^T^ (locus tag: ASILVAE211_08965) while based on MaGe annotations, a CDS encoding for a cellulase or putative endoglucanase was present in the genome of the two novel strains (*cmcAX*; EC: 3.2.1.4; ASILVAE211_v1_60021 and ACELLULO517_v1_130127) and a second CDS encoding a cellulase was identified in the genome of strain HW T2.11^T^ (EC: 3.2.1.4; ASILVAE211_v1_30210). A higher number of CDSs encoding glucosidase enzymes, potentially involved in cellobiose hydrolyse, was identified in the genome of *A. cellulosilytica* (7 CDSs) compared to *A. silvae* (2 CDSs) (Table S1.3). In addition, the cellulose biosynthesis pathway via the cellulose synthase was present in both genomes and 5 CDSs coding for the cellulose synthase operon protein C, D and YhjQ and the cellulose synthase catalytic domain [UDP-forming]/Cyclic di-GMP-binding domain (*acsAB* gene) have been identified. One may hypothesize that this signaling pathway could work in reverse direction towards cellulose hydrolysis. Overall, the two novel strains HW T2.11^T^ (*A. silvae*) and HW T5.17^T^ (*A.* *cellulosilytica*) have the genomic and physiological potential to hydrolyze cellulose and therefore to actively participate to wood degradation and to the recycling of organic matter in forest ecosystems. To date, only Murray and Woodward, [21] have shown the ability of decaying wood-inhabiting bacterial isolates to degrade cellulose. However, the taxonomic characterization of the bacterial isolates was not performed but it was demonstrated that proportion of isolates capable of degrading cellulose increased with Sitka spruce stump age [21].

#### 3.4.5. PHA Production Pathway

The PHA production pathway was also identified in both genomes by identifying the CDSs encoding acetoacetyl CoA reductase (*phaB*; EC: 1.1.1.36) and poly(3-hydroxy-alkanoate) polymerase (*phaC*; EC: 2.3.1.). For both strains, annotation with PGAP identified the 2 CDSs evidenced with MaGe (*phaB*; EC: 1.1.1.36; locus tag: ASILVAE211_01395 and ACELLULO517_03000, and *phaC* ASILVAE211_14775 and ACELLULO517_15090) but also 3 other CDSs represented by the polyhydroxyalkanoate synthesis repressor *phaR* (ASILVAE211_03430 and ACELLULO517_23745) and 2 phasin proteins (ASILVAE211_13750/ASILVAE211_01290 and ACELLULO517_03105/ACELLULO517_07245). The presence of these genes suggests that it is the class IV PHA synthase operon that is at work in these two strains. This operon is known to be widespread in bacteria belonging to the genus *Bacillus* and targets short-chain length monomers (3-hydroxybutyrate (C4) and 3-hydroxyvalerate (C5)) for polymerization [58].

## 4. Conclusions

In this study, we described two novel species belonging to the *Acidisoma* genus by combining morphological, physiological, chemotaxonomic, phylogenetic, and genomic features. The two type strains, *A. silvae* (HW T2.11^T^) and *A.* *cellulosilytica* (HW T5.17^T^) are both mesophilic and can grow in acidic environments such as decaying wood. Genetic predictions indicate that the cyclopropane fatty acid (CFA) pathway is present, conferring acid-resistance properties to the cells. These strains are able to use a wide range of organic compounds such as carbohydrates and carboxylic acids (the latter only for HW T5.17^T^) as well as to hydrolyze complex biopolymers such as starch, fucoidan, laminarin, pectin, and cellulose, the two latter being involved in wood composition. Enzymes such as cellulases and glucosidases, involved in cellulose degradation, are identified in the genome of each strain. The type strains produce storage granules of poly(3-hydroxybutyrate) (P3HB) that confer resistance to stress conditions. They might possess a class IV PHA synthase operon that is involved in the PHA production pathway. Overall, the polyphasic taxonomic analysis shows that these two novel strains are adapted to harsh environments such as decaying wood where the organic matter is difficult to access and can contribute to the degradation of dead wood.

### 4.1. Description of Acidisoma silvae sp. nov.

*Acidisoma silvae* (sil’vae L. gen. n. *silvae* from forest).

Cells of *A. silvae* are motile Gram-negative coccobacilli, 0.8–3.7 µm long, 0.4–1.0 µm wide, that occur singly but more frequently in pairs or chains and that divide by binary fission. Colonies are circular with regular edges and smooth surface with color that varies from white to pearly when cultivated on 1/10 TSA at pH 5. Cells are aerobic but are capable of growing slowly under anaerobic conditions. They are catalase-positive and oxidase-negative. They produce poly-3-hydroxybutyrate (P3HB). Cells grow at 8–25 °C (optimum, 20 °C), at pH 2.0–6.5 (optimum, pH 3.0–4.0) and with NaCl concentrations ranging between 0.0 to 2.5% (optimum, 0.5% NaCl). This type strain is chemoorganoheterotroph using a wide range of organic substrates. Growth is observed on the following C sources: sorbitol, inositol, glycerol, sucrose, mannose, trehalose, asparagine, yeast extract, and casamino acids. On the contrary, the strain is unable to use lactose (oxidative and fermentative), glucose (oxidative and fermentative), maltose, cellobiose, mannitol, ethanol, pyruvate, benzoate, citrate, lactate, butyrate, alanine, proline, histidine, asparagine, and gelatine. The strain is capable of hydrolyzing starch, fucoidan, laminarin, pectin and cellulose but is unable to hydrolyze xylan and tween 80. The following enzymatic activities were revealed: urease, arginine dihydrolase, lysine and ornithine decarboxylase, and nitrate reductase. The major cellular fatty acid is cyclo C_19: 0_*ω8c* and the major quinone is Q-10. Diphosphatidylglycerol, phosphatidylglycerol, phosphatidylcholin, phosphatidylethanolamine, an unknown aminolipid, and an unknown phospholipid are present. The GC content of the type strain is 62.50%. The 16S rRNA gene sequence and the assembled genome sequences of strain HW T2.11^T^ have been deposited into GenBank under the accession numbers MW463052 and JAESVB000000000, respectively.

The type strain HW T2.11^T^ (DSM 111006^T^; UBOCC-M-3364^T^) was isolated from the heartwood of decaying oak placed for nine months on the floor of the Champenoux forest experimental site (France).

### 4.2. Description of Acidisoma cellulosilytica sp. nov.

*Acidisoma cellulosilytica* (cel.lu.lo.si.ly’ti.ca. N.L. n. *cellulosum* cellulose; N.L. adj. *lyticus* dissolving; N.L. fem. adj. *cellulosilytica* cellulose-dissolving).

Cells of *A.* *cellulosilytica* are non-motile Gram-negative coccobacilli, 0.9–3.0 µm long, 0.6–1.0 µm wide. Cells occur singly but more frequently in pairs or chains. Colonies are circular with regular edges and smooth surface, from white to pearly in color when cultivated on 1/10 TSA at pH 5. Cells are aerobic but are capable of growing poorly under anaerobic conditions. They are catalase-positive and oxidase-negative and produce poly-3-hydroxybutyrate (P3HB). Cells grow at 14–25 °C (optimum, 20 °C), at pH 3.0–5.5 (optimum, pH 4.0–5.0) and with NaCl concentrations ranging between 0.0 to 2.5% (optimum, 0.5% NaCl). This type strain is chemoorganoheterotroph using a wide range of organic substrates. The following C sources are used: sorbitol, inositol, glucose, lactose, trehalose, sucrose, cellobiose, glycerol, ethanol, alanine, asparagine, yeast extract, and casamino acids. On the contrary, the strain is unable to use lactose (fermentative), glucose (fermentative), maltose, mannose, mannitol, pyruvate, lactate, benzoate, citrate, butyrate, proline, histidine, and gelatine. The strain is capable of hydrolyzing starch, fucoidan, laminarin, tween 80, pectin and cellulose, but is unable to hydrolyze xylan. Enzymatic activities revealed arginine dihydrolase and lysine and ornithine decarboxylases. The major cellular fatty acid is cyclo C_19: 0_*ω8c* and the major quinone is Q-10. Diphosphatidylglycerol, phosphatidylglycerol, phosphatidylcholin, phosphatidylethanolamine, phosphatidylserine, an unknown aminolipid, and an unknown phospholipid are present. The GC content is 62.32%. The 16S rRNA gene sequence and the assembled genome sequences of strain HW T5.17^T^ have been deposited in GenBank under the accession numbers MW463051 and JAESVA000000000, respectively.

The type strain HW T5.17^T^ (DSM 111007^T^; UBOCC-M-3365^T^) was isolated from the heartwood of decaying oak placed for nine months on the floor of the Champenoux forest experimental site (France).

## Figures and Tables

**Table 1:** Characteristics differentiating strains HW T2.11^T^ (*Acidisoma silvae*) and HW T5.17^T^ (*A.* *cellulosilytica*) from other species of the genus *Acidisoma*. Characteristics are scored as: +, positive; −, negative; V, variable. The four strains utilized sorbitol, inositol, sucrose, glycerol, yeast extract and casamino acids. None of the strains was able to utilize lactose (fermentative), glucose (fermentative), maltose, mannitol, benzoate, citrate, butyrate, and gelatin. They were all capable of hydrolyzing starch, fucoidan, laminarin, and pectin.

Characteristics	Str. HW T2.11^T^	Str. HW T5.17^T^	*A. tundrae* (WM1^T^)	*A. sibiricum* (TPB606^T^)
Colony color	White, cream, pearly	White, ivory, pearly	White, ivory (*)	White, cream, pinkish (*)
Oxidase	−	−	+	+
Cell size (µm)	0.4–1.0 × 0.8–3.7	0.6–1.0 × 0.9–3.0	0.8–1.5 × 1.9–4.1 (*)	0.7–1.2 × 1.4–3.1 (*)
Cell shape	coccobacilli	coccobacilli	Coccobacilli	coccobacilli
Motility	+	−	−	−
Temperature range (optimum) (°C)	8–25 (20)	14–25 (20)	2–30 (15–22) (*)	2–30 (20–25) (*)
pH growth range (optimum) (°C)	2.0–6.5 (3.0–4.0)	3.0–5.5 (4.0–5.0)	3.0–7.5 (4.5–5.7) (*)	3.7–7.6 (5.0–6.5) (*)
NaCl tolerance (optimum) (%, *w*/*v*)	0.0–2.5 (0.5)	0.0–2.5 (0.5)	<1.5 (*)	<2.5 (*)
Carbon source utilization				
Glucose	−	+	+	+
Lactose	−	+	+	+
Mannose	+	−	+	+
Trehalose	+	+	+	+
Ethanol	−	+	−	−
Pyruvate	−	−	+	+
Lactate	−	−	−	+
Asparagine	V	V	+	+
Alanine	− (†)	+ (†)	− (*)	−(*)
Proline	− (†)	− (†)	+ (*)	+ (*)
Histidine	− (†)	− (†)	+ (*)	+ (*)
Cellobiose	− (†)	+ (†)	− (*)	V (*)
Carboxymethylcellulose	+	+	–	−
Xylan	− (†)	− (†)	+ (*)	+ (*)
Tween 80	− (†)	+ (†)	− (*)	− (*)
Enzymatic activities				
Urease	+	−	−	+
Arginine dihydrolase	+	+	− (*)	− (*)
Nitrate reductase	+	−	−	−

† From Mieszkin et al. [17]; * From Belova et al. [25].

**Table 2:** Whole-cell fatty acid profiles (% of the total) of strains HW T2.11^T^ (*Acidisoma silvae*), HW T5.17^T^ (*A.* *cellulosilytica*) and previously described type strains of *A. tundrae* (WM1^T^) and *A. sibiricum* (TPB606^T^). Values are percentages of the fatty acids that were assigned to fatty acids in the peak-naming table of the MIS database (MIDI, Microbial ID, Newark, DE 19711 U.S.A.). The nomenclature is as follow: the first number indicates the number of carbon atoms in the molecule; ‘iso’, ‘OH’, ‘methyl’ and ‘cyclo’ indicate isobranched, hydroxy, methylated or cyclic fatty acids; the second number following the colon indicates the number of double bonds present. The position of the double bond is indicated by the carbon atom position starting from the methyl (*ω*) end of the molecule. *c*, cis isomer. Major fatty acids are indicated in bold.

Fatty Acids	Strain HWT2-11^T^	Strain HWT5-17^T^	*A. tundrae* (WM1^T^)	*A. sibiricum* (TPB606^T^)
C_10: 0_	-	-	0.1	-
C_12:0_	-	-	0.2	-
C_14: 0_	-	-	0.2	0.7
Unknown 14.959	2.1	2.1	3.1	1.4
Summed feature 2 *	1.0	0.9	1.9	2.0
Summed feature 3 †	0.5	0.4	-	1.7
C_16: 0_ N alcohol	-	-	0.4	-
C_16: 1_*ω5c*	-	-	-	0.2
C_16: 0_	6.3	6.6	4.7	21.3
C_16: 0_ 2-OH	1.1	1.9	-	0.5
C_16: 0_ 3-OH	0.5	0.5	0.2	1.2
Cyclo C_17: 0_	-	-	-	1.2
C_17: 0_ 3-OH	-	-	0.4	-
C_17: 0_	-	0.3	-	-
C_18: 1_*ω9c*	-	-	2.4	0.7
C_18: 1_*ω7c*	16.3	7.4	11.1	13.8
C_18: 1_*ω5c*	-	0.7	0.4	-
C_18: 0_	2.9	3.9	6.0	2.9
11-Methyl C_18: 1_*ω*7*c*	0.9	1.2	1.2	0.8
C_18: 1_ 2-OH	5.9	8.8	-	0.8
C_18: 0_ 3-OH	7.6	8.0	10.7	5.6
Cyclo C_19: 0_*ω8c*	53.9	55.8	55.4	44.4
C_20: 2_ *ω*6,9*c*	1.0	1.4	1.4	1.1

-, Not detected; * Summed feature 2 comprises iso-C16: 1 and/or C14: 0 3-OH. † Summed feature 3 comprises C16: *ω*7c and/or iso-C15: 0 2-OH.

**Table 3:** Genomic features of strains HW T2.11^T^ 11^T^ (*Acidisoma silvae*) and HW T5.17^T^ (*A.* *cellulosilytica*).

Genomic Features	HW T2.11^T^	HW T5.17^T^
Number of contigs	82	59
Genome size (bp)	5,444,225	6,010,181
Longest contig (bp)	974,443	922,216
*N_50_*	540,961	572,597
*N_75_*	112,774	177,353
*L_50_*	4	4
*L_75_*	9	9
GC content (%)	62.50	62.32
CDS	5501	6045
RNAs	48	58
tRNAs	45	52
16S-23S-5S rRNAs	1-1-1	2-2-2

## Supplementary Materials

The following are available online at https://www.mdpi.com/article/10.3390/microorganisms9102053/s1. Table S1.1: Characteristics differentiating strain HW T2.11^T^ (*Acidisoma silvae*) from strain HW T5.17^T^ (*A.* *cellulosilytica*) using Biolog GN2 microplate from Mieszkin et al. [17]; Table S1.2: Classification of the CDS of HW T2.11^T^ (*Acidisoma silvae*) and HW T5.17^T^ (*A.* *cellulosilytica*) in COG categories; Table S1.3: Identification of enzymes involved in cellulose hydrolysis in the genome of strains HW T2.11^T^ (*Acidisoma silvae*) and HW T5.17^T^ (*A. cellulosilytica*) based on genes homology using PGAP and MaGe pipelines; Figure S1.1: Scheme of the experimental procedure; Figure S1.2: Growth kinetics of strains HW T2.11^T^ (*Acidisoma silvae*) and HW T5.17^T^ (*A.* *cellulosilytica*); Figure S1.3: Location of the 4156 georeferenced records (among the total 4423 occurrences) for the genus *Acidisoma* in July 2021 based on the metagenomic 16S ribosomal RNA gene sequences from the GBIF database (https://www.gbif.org/species/8071706 (accessed on 1 July 2021)); Figure S1.4: Circular mapping of the genome of strains (a) HW T2.11^T^ (*Acidisoma silvae*) and (b) HW T5.17^T^ (*A.* *cellulosilytica*) obtained from the circular genome viewer of the MaGe platform.

## Abbreviations

ANI	Average Nucleotide Identity
CDS	Coding DNA Sequences
CFA	Cyclopropane fatty acids
CMC	Carboxymethylcellulose
dDDH	Digital DNA-DNA hybridization
DNA	Deoxyribonucleic acid
DMSO	Dimethyl sulfoxide
DSMZ	Deutsche Sammlung von Mikroorganismen und Zellkulturen
FTIR	Fourier Transform Infrared
GGDC	Genome-to-genome distance calculator
KEGG	Kyoto Encyclopedia of Genes and Genomes
MaGe	Microbial Genome Annotation and Analysis Platform
MMN	Mobility-Mannitol-Nitrate
NMR	Nuclear Magnetic Resonance
NCBI	National Center for Biotechnological Information
OGRI	Overall Genome Relatedness Indices
OrthoANI	Orthologous Average Nucleotide Identity
PCI	Phenol Chloroform Isoamyl Alcohol
PHAs	Polyhydroxyalcanoates
PHB	Polyhydroxybutyrate
PGAP	Prokaryotic genome annotation pipeline
RC	Red Congo
rRNA	Ribosomal ribonucleic acid
SEM	Scanning Electron Microscopy
TCA	Tricarboxylic Acid cycle
TEM	Transmission Electron Microscopy
TSA	Tryptic Soy Agar
TSB	Tryptic Soy Broth
UBOCC	UBO Culture Collection
