# Supplementary material for: Acidisoma silvae sp. nov. and Acidisoma cellulosilytica sp. nov., Two Acidophilic Bacteria Isolated from Decaying Wood, Hydrolyzing Cellulose and Producing Poly-3-hydroxybutyrate
Source: Microorganisms. 2021 Sep 28;9(10):2053. doi: 10.3390/microorganisms9102053 (PMC8537097; doi:10.3390/microorganisms9102053)
Supplement: Supplementary file 1 [file microorganisms-09-02053-s001.zip › microorganisms-1377238-SI.pdf]

Supplementary Materials

Table S1.1: Characteristics differentiating strain HW T2.11^T (*Acidisoma silvae*) from strain HW T5.17^T (*A. cellulosilytica*) using Biolog GN2 microplate (adapted from Mieszkin *et al.* [17]). Characteristics are scored as: +, positive or –, negative.

Concerning the amino acids category, the two strains utilized L-Alanine and L-Asparagine but neither strain was capable of utilized L-Histidine, Hydroxy-L-Proline, Inosine, L-Ornithine, L-Phenylalanine, L-Proline, L-Alanyl-glycine, D-Serine, L-Serine, D.L-Carnitine and L-Glutamic Acid.

Concerning the amines/amides category, the two strains utilized L-Alaninamide and but neither strain was capable of utilized Phenyethylamine, Putrescine, Glucuronamide, L-Threonine and 2-Aminoethanol.

Concerning the carbohydrates category, the two strains utilized i-Erythritol, D-Fructose, Dextrin, L-Fucose, D-Galactose, Alpha-D-Glucose, D-Sorbitol, D-Trehalose, Adonitol, D_Mannose, L-Arabinose, Xylitol, and D-Arabitol but neither strain was capable of utilized Gentiobiose, L-Rhmanose, Alpha-D-Lactose, D-Mannitol, D-Melibiose, Beta-Methyl-D-Glucoside, D-Psicose, D-Raffinose, Sucrose, N-Acetyl-D-glucosamine, Lactulose, Turanose and Maltose.

Concerning the carboxylic acids category, the two strains utilized D-L-Lactic Acid and D-Gluconic-Acid but neither strain was capable of utilized Urocanic-Acid, propionic acid, Glycyl-L-Glutamic Acid, Acetic Acid, p-Hydroxy-Phenylacetic-Acid, Bromo-Succinic-Acid, *Cis*-Aconitic-Acid, Itaconic-Acid, Succinamic-Acid, Citric-Acid, Alpha-Keto-Butyric-Acid, Formic-Acid, Alpha-Keto-Glutaric-Acid, Alpha-Keto-Valeric-Acid, D-Galacturonic-Acid, Malonic-Acid, L-Pyroglutamic-Acid, L-Aspartic-Acid, Quinic-Acid, Sebacic-Acid and Glycyl-L-Aspartic-Acid.

Concerning the miscelleanous category, the two strains utilized Glycreol, Mono-Methyl-Succinate and glucose-6-Phosphate but neither strain was capable of utilized Glucose-1-Phosphate, Uridine, Glycogen, Thymidine, 2-Aminoethanol, 2.3-Butanediol and D.L-Alpha-Glycerol-Phosphate.

Concerning the polymer category, none of the strain was capable of hydrolyzing Alpha-Cyclodextrin.

		HW T2.11T	HW T5.17T
**Amino acids**			
	L Leucine	+	-
	D Alanine	-	+
**Amines/Amides**			
	N-Acetyl-D-galactosamine	-	+
**Carbohydrates**			
	L Fucose	-	+
	m-Inositol	-	+
	D cellobiose	-	+
**Carboxylic acids**			
	D-Glucosaminic-Acid	-	+
	D-Glucuronic-Acid	-	+
	Alpha-Hydroxy-Butyric-Acid	-	+
	D-Saccharic-Acid	-	+
	Beta-Hydroxy-Butyric-Acid	-	+
	Gamma-Hydroxy-Butyric-Acid	-	+
	Succinic-Acid	-	+
	Gamma-Amino-Butyric-Acid	-	+
	D-Galactonic-Acid-Lactone	-	+
**Polymers**			
	Tween 40	-	+
	Tween 80	-	+
**Miscelleanous**			
	Methyl-Pyruvate	-	+

Table S1.2: Classification of the CDS of strain HW T2.11^T (*Acidisoma silvae*) and strain HW T5.17^T (*A. cellulosilytica*) in COG categories.

			HW T2.11	HW T2.11	HW T5.17	HW T5.17
**Processes**	**Class ID**	**Description**	**CDS (nb)**	**CDS (%)**	**CDS (nb)**	**CDS (%)**
**CELLULAR PROCESSES AND SIGNALING**	D	Cell cycle control, cell division, chromosome partitioning	50	0.9089	46	0.761
	M	Cell wall/membrane/envelope biogenesis	293	5.3263	297	4.9132
	N	Cell motility	69	1.2543	49	0.8106
	O	Posttranslational modification, protein turnover, chaperones	147	2.6722	165	2.7295
	T	Signal transduction mechanisms	194	3.5266	165	2.7295
	U	Intracellular trafficking, secretion, and vesicular transport	95	1.727	92	1.5219
	V	Defense mechanisms	39	0.709	44	0.7279
	W	Extracellular structures	6	0.1091	7	0.1158
**INFORMATION STORAGE AND PROCESSING**	B	Chromatin structure and dynamics	4	0.0727	5	0.0827
	J	Translation, ribosomal structure and biogenesis	197	3.5812	209	3.4574
	K	Transcription	434	7.8895	508	8.4036
	L	Replication, recombination and repair	266	4.8355	265	4.3838
**METABOLISM**	C	Energy production and conversion	278	5.0536	310	5.1282
	E	Amino acid transport and metabolism	628	11.4161	696	11.5136
	F	Nucleotide transport and metabolism	91	1.6542	93	1.5385
	G	Carbohydrate transport and metabolism	520	9.4528	668	11.0505
	H	Coenzyme transport and metabolism	152	2.7631	155	2.5641
	I	Lipid transport and metabolism	231	4.1992	262	4.3342
	P	Inorganic ion transport and metabolism	331	6.0171	365	6.038
	Q	Secondary metabolites biosynthesis, transport and catabolism	206	3.7448	259	4.2845
**POORLY CHARACTERIZED**	R	General function prediction only	758	13.7793	857	14.177
	S	Function unknown	307	5.5808	347	5.7403

Table S1.3: Identification of enzymes involved in cellulose hydrolysis in the genome of strains HW T2.11^T (*Acidisoma silvae*) and HW T5.17^T (*A. cellulosilytica*) based on genes homology using PGAP and MaGe pipelines.

	Acidisoma silvae (HW T2.11^T)		*Acidisoma cellulosilytica* (HW T5.17^T)	
**Enzymes**	**PGAP gene associated locus/EC number**	**MaGe gene associated locus/EC number**	**PGAP gene associated locus/EC number**	**MaGe gene associated locus/EC number**
**Cellulase**	ASILVAE211_08965	ASILVAE211_v1_30210/EC 3.2.1.4		
**Putative endoglucanase or cellulase (*cmcAX*)**		ASILVAE211_v1_60021/EC 3.2.1.4		ACELLULO517_v1_130127/EC 3.2.1.4
**Glucosidase**	ASILVAE211_00975		ACELLULO_03405	
**Amylo-α-1,6-glucosidase**	ASILVAE211_03165	ASILVAE211_v1_10655	ACELLULO_23510	ACELLULO517_v1_120018
**β-glucosidase**			ACELLULO_11860/EC 3.2.1.23	
**β-glucosidase A**				ACELLULO517_v1_30664/EC 3.2.1.21
**α-glucosidase or α-galactosidase**			ACELLULO_14440<br>ACELLULO_16405<br>ACELLULO_20735	

Figure S1.1: Scheme of the experimental research procedure.

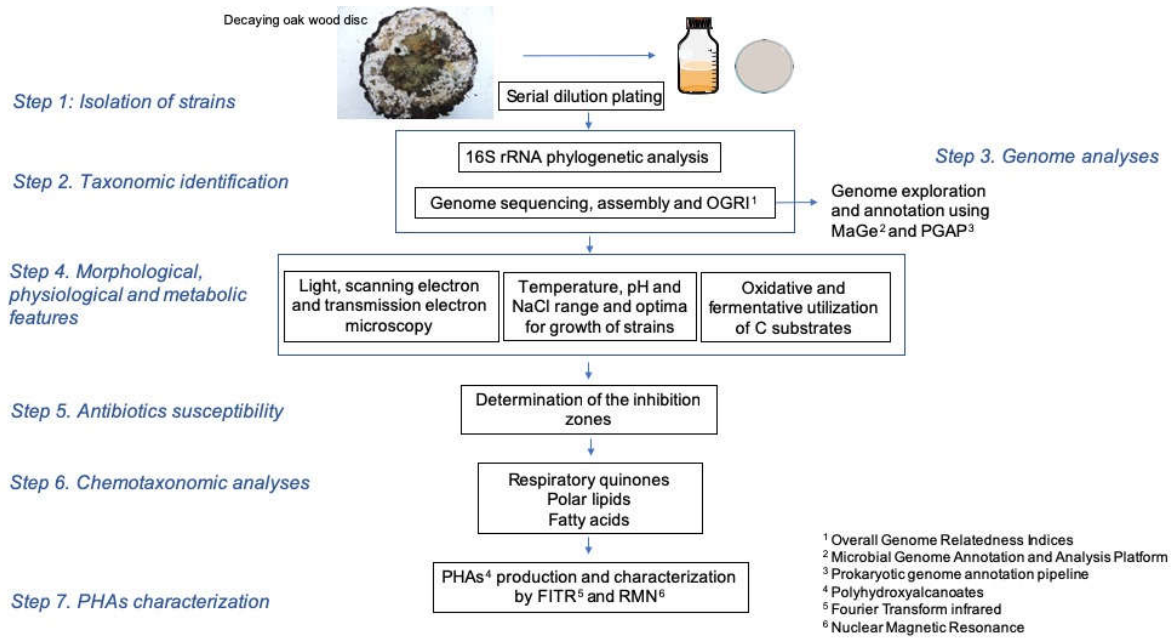

Figure S1.2: Growth kinetics under optimal growth conditions of strains HW T2.11^T (*Acidisoma silvae*) and HW T5.17^T (*A. cellulosilytica*).

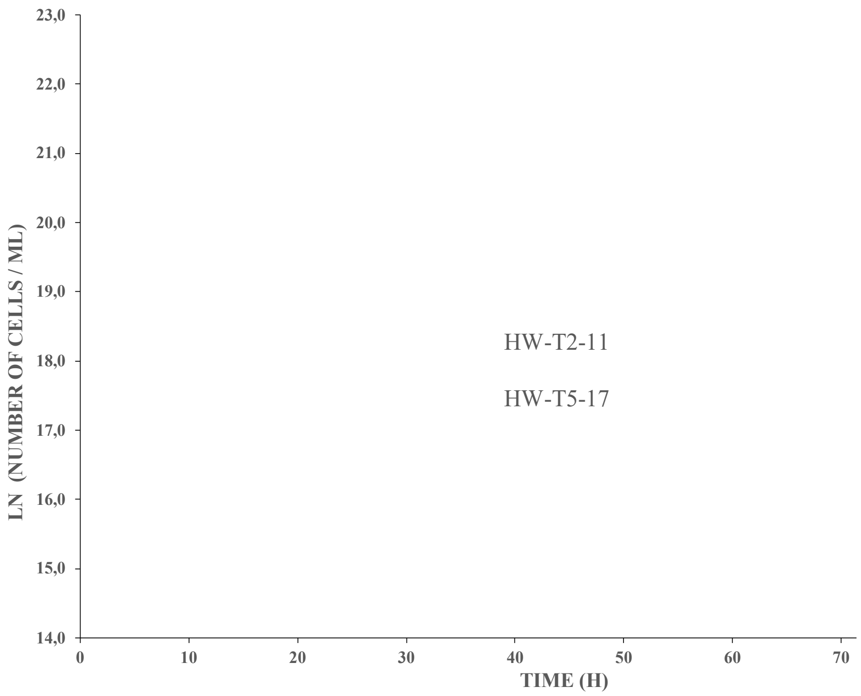

Figure SI.3: Location of the 4,156 georeferenced records (among the total 4,423 occurrences) for the genus *Acidisoma* in July 2021 based on the metagenomic 16S ribosomal RNA gene sequences from the GBIF database (https://www.gbif.org/species/8071706).

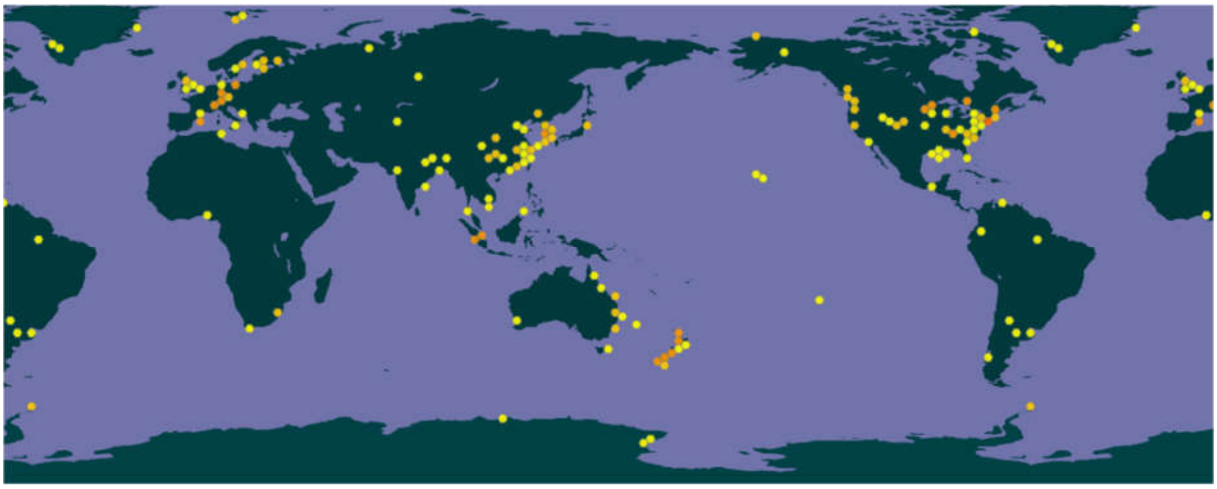

Figure S1.4: Circular mapping of the genome of strains (a) *Acidisoma silvae* (HW T2.11^T) and (b) *A. cellulosilytica* (HW T5.17^T) obtained from the circular genome viewer of the MaGe platform.

a.

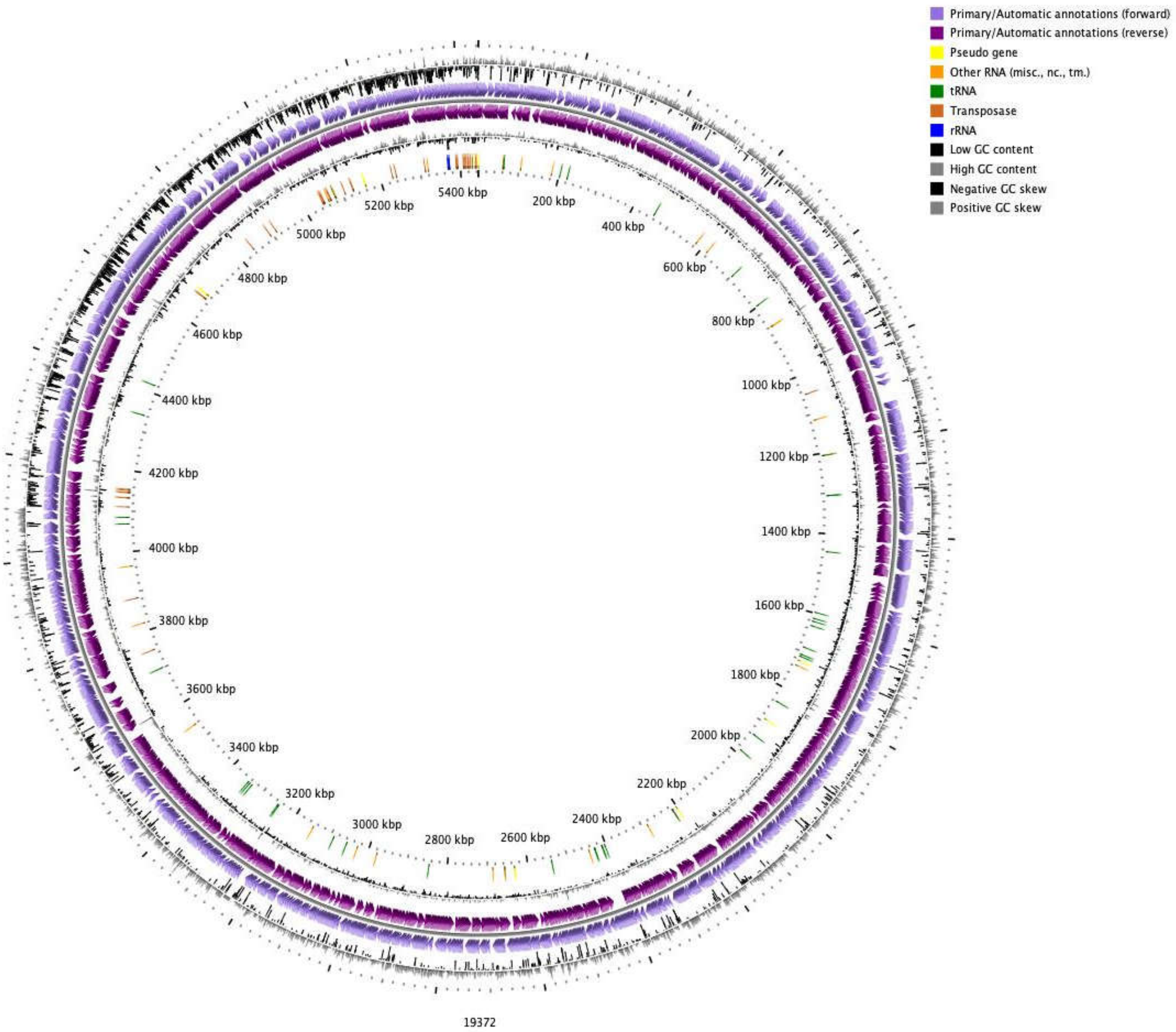

b.

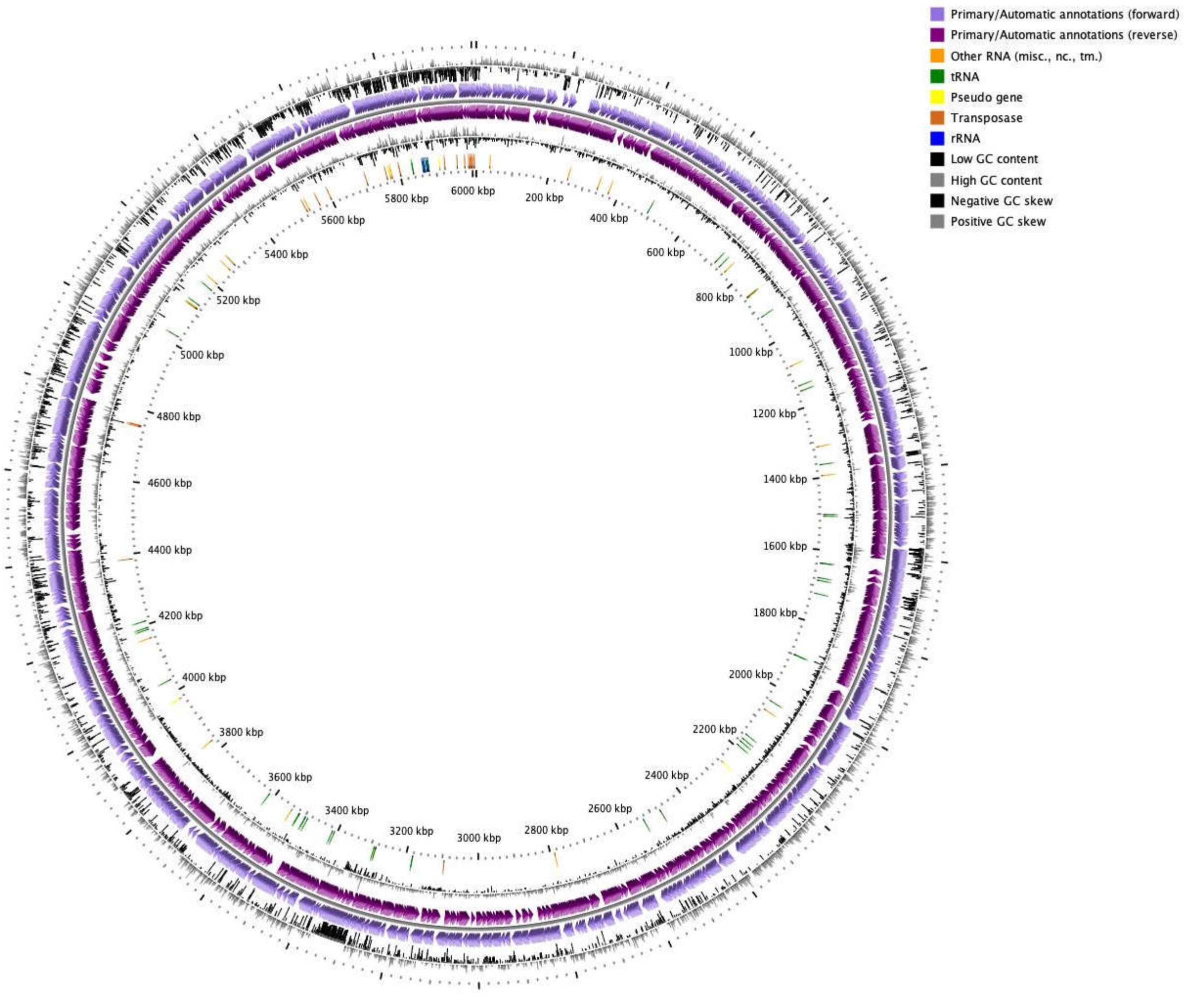

Primary/Automatic annotations (forward)
Primary/Automatic annotations (reverse)
Other RNA (misc., nc., tm.)
tRNA
Pseudo gene
Transposase
rRNA
Low GC content
High GC content
Negative GC skew
Positive GC skew
6000 kbp
200 kbp
400 kbp
600 kbp
800 kbp
1000 kbp
1200 kbp
1400 kbp
1600 kbp
1800 kbp
2000 kbp
2200 kbp
2400 kbp
2600 kbp
2800 kbp
3000 kbp
3200 kbp
3400 kbp
3600 kbp
3800 kbp
4000 kbp
4200 kbp
4400 kbp
4600 kbp
4800 kbp
5000 kbp
5200 kbp
5400 kbp
5600 kbp
5800 kbp

19371
